# Semantic network from the words happiness and wellbeing: Dataset in a Mexican sample

**DOI:** 10.1016/j.dib.2019.104830

**Published:** 2019-11-16

**Authors:** Maria F. Durón-Ramos, Fernanda I. García-Vazquez, Luis M. Castellanos

**Affiliations:** a Instituto Tecnológico de Sonora, Department of Pshychology, Guaymas, Sonora, Mexico; b Instituto Tecnológico de Sonora, Department of Education, Obregón, Sonora, Mexico; c Universidad de la frontera, Center of Excellence in Economic and Consumer Psychology, Temuco, Chile

**Keywords:** Happiness, Wellbeing, Mexico, Semantic network

## Abstract

The present paper presents data with information about related words for happiness and wellbeing attributed by people living in a northern city of Mexico. Quota sampling technique was used to collect the data following the distribution of the population by age and gender. National Institute of Geography and Statistics in México was consulted to obtain the proportion of age range and gender in the city of Guaymas, Sonora. Participants from 18 to 85 years old (M = 41.77, ST = 16.76) answer the instrument for a semantic network technique, which is composed by two steps, first freely answer words related to each concept (happiness/wellbeing), and second, hierarchizing them from the most to the least related. The dataset presents the words given by the participants ordered by their hierarchy; number one indicates the concept that people indicated as the most related to the concept. This article presents the ten more related words for each concept (Table 1), the ten related words by male and female for wellbeing (Table 2), and happiness (Table 3). Health is more related to wellbeing, while the family is more frequently related to happiness.

**Table:** Specifications Table

Subject	Social Sciences
Specific subject area	Linguistics and Language
Type of data	Excel document, table in the article
How data were acquired	Survey with questionnaire in paper was presented to the participants (Sumplementary material 1)
Data format	Raw and Analyzed
Parameters for data collection	First, the participants were asked to write between 5 and 10 words related to happiness and other 5 to 10 related with wellbeing; then, they were asked to rate them from the most related (1) to the least (10) for each concept. This technique is used to form a semantic network in order to understand how people think and feel about happiness and wellbeing.
Description of data collection	Quota sampling was used, distributing to the population by age ranges that represent different life cycle stages (from youth to old age); these percentages followed the normal distribution of the population in Guaymas, according to the National Institute of Geography and Statistic (INEGI). Gender was also distributed according to the population, which is 52% female and 48%, male.
Data source location	General population from Guaymas, Sonora México.
Data accessibility	Repository name: Mendeley data Data identification number: 10.17632/8hwr42zvh6.1 Direct URL to data: https://doi.org/10.17632/8hwr42zvh6.1

**Table:** 

**Value of the Data** • Data presented here represents the meaning attributed to happiness and wellbeing by people living in a developing country (Mexico), a population overlooked when examining these positive aspects. • These data include information from people in different age range and a well-balanced gender proportion, which can provide a better understanding of the concepts attributed to happiness and wellbeing. • Future research focused on happiness and wellbeing on a Spanish speaking population can benefit from the information provided by these data. • These data can be used to create an instrument to measure happiness and wellbeing adequately for people living in Mexico or a Latin-American country.

## 1 Data

The supplementary material 2 is a data file in excel format, each line represents a participant; columns contains the words considered related to concepts presented (wellbeing/happiness), columns are in the order that participants rated to their concept (not in the order named): first column “Wellbeing 1” holds the words that participants rated as the most related to wellbeing (with number 1), the second column “Wellbeing 2” presents the words rated as the second most related (with number 2), and so on, until “Wellbeing 10”. Same format for the words given for the happiness concept.

With this information, three essential values were calculated: a) frequency or times mention; b) semantic weight or M value, which is the frequency multiplied by the semantic value that was given according to the hierarchy of peoples rating (from 1 to 10); c) FMG value, punctuation expressed as a percentage of the defining words that make up all the words related to the concept of interest (considering the higher M value as 100%) [1,2]. These values are presented in general for both wellbeing and happiness (Table 1); and separated by gender in Table 2 (wellbeing) and Table 3 (happiness).

**Table 1:** Ten words most related to wellbeing and happiness.

	Wellbeing				Happiness			
		F	M	FMG		F	M	FMG
1	Health (Salud)	115	895	100	Family (Familia)	106	931	100
2	Happiness (Felicidad)	44	351	39	Love (Amor)	70	484	52
3	Tranquility (Tranquilidad)	49	346	39	Health (Salud)	35	271	29
4	Family (Familia)	38	335	37	Joy (Alegría)	35	251	27
5	Comfort (Comodidad)	43	266	30	Tranquility (Tranquilidad)	27	232	25
6	Economy (Economía)	37	262	29	Friends (Amigos)	35	229	25
7	Work (Trabajo)	32	209	23	Wellbeing (Bienestar)	27	217	23
8	Nourishment (Alimentación)	26	170	19	Work (Trabajo)	20	136	15
9	Love (Amor)	23	161	18	Success (Éxito)	14	103	11
10	Feeling good (Sentirse bien)	11	101	11	Economy (Economía)	14	93	10

**Table 2:** Ten words most related to wellbeing separated by gender.

	Males				Females			
		F	M	FMG		F	M	FMG
1	Health (Salud)	55	402	100	Health (Salud)	60	493	100
2	Happiness (Felicidad)	24	187	47	Family (Familia)	21	199	40
3	Tranquility (Tranquilidad)	22	164	41	Economy (Economía)	27	185	38
4	Family (Familia)	17	136	34	Tranquility (Tranquilidad)	27	182	37
5	Comfort (Comodidad)	18	113	28	Happiness (Felicidad)	20	164	33
6	Feeling good (Sentirse bien)	11	101	25	Comfort (Comodidad)	25	153	31
7	Work (Trabajo)	12	90	22	Work (Trabajo)	20	119	24
8	Nourishment (Alimentación)	11	84	21	Nourishment (Alimentación)	15	86	17
9	Love (Amor)	12	84	21	Safety (Seguridad)	11	84	17
10	Economy (Economía)	10	77	19	Love (Amor)	11	77	16

**Table 3:** Ten words most related to happiness separated by gender.

	Males				Females			
		F	M	FMG		F	M	FMG
1	Family (Familia)	51	436	100	Family (Familia)	55	495	100
2	Love (Amor)	24	198	45	Love (Amor)	46	286	58
3	Tranquility (Tranquilidad)	14	150	34	Joy (Alegría)	22	155	31
4	Wellbeing (Bienestar)	17	136	31	Health (Salud)	18	137	28
5	Health (Salud)	17	134	31	Friends (Amigos)	18	103	21
6	Friends (Amigos)	17	126	29	Economy (Economía)	14	93	19
7	Success (Éxito)	14	103	24	Tranquility (Tranquilidad)	13	82	17
8	Joy (Alegría)	13	96	22	Wellbeing (Bienestar)	10	81	16
9	Work (Trabajo)	11	74	17	Enjoy (Disfrutar)	8	65	13
10	Peace (Paz)	8	66	15	Work (Trabajo)	9	62	13

## 2 Experimental design, materials, and methods

### 2.1 Participants

137 people from Guaymas, Sonora, Mexico gave their answers. To obtain a sample that could represent the population according to peoples age and gender, a quota sampling technique was used. Percentages of age range and gender followed the normal distribution of the population in Guaymas, according to the National Institute of Geography and Statistic [3]. Five age range were considered: from 18 to 25 (19%), 26 to 34 (21%), 35 to 49 (31%), 50 to 64 (19%), over 65 (10%). Female represented 52% of the sample and male the 48%.

### 2.2 Instruments

An instrument for semantic network proposed by Vera, Pimentel y Batista [1] was used to collect the data. First, the participants were asked to write between 5 and 10 words related with happiness and other 5 to 10 related with wellbeing; second, they were asked to rate them from the most related (1) to the least (10) for each concept. This technique is an empirical method to access the subjective interpretation that a person makes of the words [4], in this case, to understand how people think and feel about happiness and wellbeing.
