# Supplementary material for: Semantic network from the words happiness and wellbeing: Dataset in a Mexican sample
Source: Data Brief. 2019 Nov 16;27:104830. doi: 10.1016/j.dib.2019.104830 (PMC6889765; doi:10.1016/j.dib.2019.104830)
Supplement: Multimedia component 2 [file mmc2.pdf]

Thank you for participating, the information you provide is completely ANONYMOUS. We inform you that there are no right and wrong answers, we just want to know your opinion of the subject.

Gender: ☐ Male ☐ Female Age: _______

Please define WELLBEING using ONLY one or two words on each line (Verbs, nouns or Adjectives).	Please define HAPPINESS using ONLY one or two words on each line (Verbs, nouns or Adjectives).
☐ ________________________________	☐ ________________________________
☐ ________________________________	☐ ________________________________
☐ ________________________________	☐ ________________________________
☐ ________________________________	☐ ________________________________
☐ ________________________________	☐ ________________________________
☐ ________________________________	☐ ________________________________
☐ ________________________________	☐ ________________________________
☐ ________________________________	☐ ________________________________
☐ ________________________________	☐ ________________________________
☐ ________________________________	☐ ________________________________

Finally, please order the list of words you made by putting the number 1 to what you consider the most important, the most related, close, or that best defines well-being and happiness (respectively), then the number 2 and so on until finish ranking each and every word listed.

Thank you!
